# Medical management of acute heart failure

**DOI:** 10.12703/r/10-82

**Published:** 2021-12-06

**Authors:** Hayaan Kamran, W H Wilson Tang

**Affiliations:** 1 Department of Cardiovascular Medicine, Heart, Vascular and Thoracic Institute, Cleveland Clinic, Cleveland, OH, USA

**Keywords:** acute heart failure, diagnosis, management, diuretic resistance, goal directed medical therapy

## Abstract

Despite recent advances in the treatment of chronic heart failure, therapeutic options for acute heart failure (AHF) remain limited. AHF admissions are associated with significant multi-organ dysfunction, especially worsening renal failure, which results in significant morbidity and mortality. There are several aspects of AHF management: diagnosis, decongestion, vasoactive therapy, goal-directed medical therapy initiation and safe transition of care. Effective diagnosis and prognostication could be very helpful in an acute setting and rely upon biomarker evaluation with noninvasive assessment of fluid status. Decongestive strategies could be tailored to include pharmaceutical options along with consideration of utilizing ultrafiltration for refractory hypervolemia. Vasoactive agents to augment cardiac function have been evaluated in patients with AHF but have shown to only have limited efficacy. Post stabilization, initiation of quadruple goal-directed medical therapy—angiotensin receptor-neprilysin inhibitors, mineral receptor antagonists, sodium glucose type 2 (SGLT-2) inhibitors, and beta blockers—to prevent myocardial remodeling is being advocated as a standard of care. Safe transition of care is needed prior to discharge to prevent heart failure rehospitalization and mortality. Post-discharge close ambulatory monitoring (including remote hemodynamic monitoring), virtual visits, and rehabilitation are some of the strategies to consider. We hereby review the contemporary approach in AHF diagnosis and management.

## Introduction

Acute heart failure (AHF) is a complex heterogeneous clinical syndrome associated with cardiac dysfunction leading to pulmonary and systemic congestion or hypoperfusion or both. Increasingly, hospital admissions or emergency department visits rather than physiologic manifestations, are the metrics being used to assess the clinical outcomes of AHF patients. Novel drugs are being investigated to mitigate the physiological burden of AHF hospitalizations. Appropriate pharmaceutical interventions are important, as prevalence of AHF is increasing with the aging population in the US, where there are more than one million hospitalizations per year^1^. AHF is also associated with significant readmission rates exceeding 25% at 3 months and 50% at 6 months^2^. The 5-year mortality rate remains exceptionally high at 50 to 60%^1,2^. This imposes a significant financial burden with a projected increase in health-care costs to $78 billion by the end of this decade^3^. The majority of AHF cases are indeed preventable and hence more effort should be put forth toward applying disease-modifying therapy both before and during AHF hospitalizations, a period that can be viewed as a continuum of the natural history of heart failure (HF). Timely initiation helps prevent cardiac remodeling and disease progression of HF. We hereby critically review the major developments that have shaped our contemporary diagnostic and therapeutic strategies in AHF.

## Under-utilization of biomarkers in acute heart failure

For years, natriuretic peptides (NPs) have been the cornerstone for AHF diagnosis and prognostication. In the setting of volume and pressure overload, NPs are produced by dilated ventricles, which are catabolized into active B-type NP (BNP) and an inactive more stable form, N-terminal-proBNP (NT-proBNP). NPs have a high negative predictive value for AHF, and established cutoffs are less than 100 pg/mL and less than 300 ng/mL for BNP and NT-proBNP, respectively^4^. No one type of NP is proven to be superior over another, and quantitatively they are not equivalent but are largely concordant. Among patients taking angiotensin receptor-neprilysin inhibitors (ARNIs), NT-proBNP levels are theoretically more useful than BNP levels as the former have a longer half-life and are unaffected by neprilysin inhibition^5^. In morbidly obese patients with AHF, a lower cutoff for NPs should be considered given that these patients tend to have low NP levels despite being in HF^6,7^. In contrast, among elderly patients, higher cutoffs have been proposed^8^. NPs also continue to be an effective prognostic marker in HF patients with renal dysfunction, but higher cut points maybe more useful because of their decreased excretion^9^. There is also an important prognostic role of NP in patients presenting with AHF when measured at admission (class I indication) or even at the time of discharge (class IIa indication)^10^. Higher levels of NPs on admission are associated with an independent all-cause risk for cardiovascular (CV) mortality and morbidity^11^. Decreases in NPs during admission and pre-discharge NP levels are also associated with decrease in mortality and recurrent hospitalization^12^. No universal cutoffs have been shown to predict improved outcomes, although the consensus is that a more than 30% reduction in NP levels portends a better overall prognosis^13^.

Cardiac troponins (both troponin T or I) are also released in the setting of AHF exacerbation, which in the absence of acute coronary syndrome, have been associated with an independent risk of mortality and readmission^13^. Temporal reductions in troponin levels through the hospitalization have also been associated with the risk mitigation of adverse events^14^. The high-sensitivity troponins were also evaluated in a prospective observational study and were not found to be a useful marker for risk stratification of AHF patients who were at high risk of hospitalization and mortality^15^. Novel inflammatory biomarkers such as soluble sT2, a soluble form of interleukin 1 (IL-1) receptor-like 1 and IL-1β, are also being evaluated to prognosticate AHF-related mortality and rehospitalization^16^. Higher levels of galectin 3 (a biomarker of myocardial fibroblast activation) and soluble ST2 (a biomarker of myocardial stretch) have also been associated with adverse short-term events, including mortality in patients with AHF^17,18^. GlycA, a nuclear magnetic composite marker of systemic inflammation, which is associated with glycosation state of main acute phase reactants, has been shown to be associated with increased risk of developing any HF in particular HF with preserved ejection fraction (HFpEF)^19^ Another marker that has prospectively been shown to be promising in AHF patients, especially in setting of renal dysfunction, is plasma carbohydrate antigen 125 (CA125). CA125 is a surrogate marker of fluid overload, which, when prospectively used to guide diuresis, has been shown to significantly improve diuresis and renal function at 72 hours^20^. Further studies are needed to elucidate the role of CA125 in decongestive therapies. These biomarkers, in combination with NPs, could further help prognosticate and risk-stratify patients with AHF^21^. In summary, other factors such as left ventricular ejection fraction (EF) have failed to demonstrate prognostic utility beyond that of NPs or cardiac troponins^22^. Revised guidelines published by the ACC/AHA/HFSA (American College of Cardiology/American Heart Association/Heart Failure Society of America) in 2017 recommend the use of NPs and troponins for diagnosis and risk stratification of patients presenting with AHF exacerbation. Pre-discharge measurement of levels of NPs was also recommended for prognostication of post-discharge course^10^.

## Better assessments of volume and perfusion status

**Figure 1.**
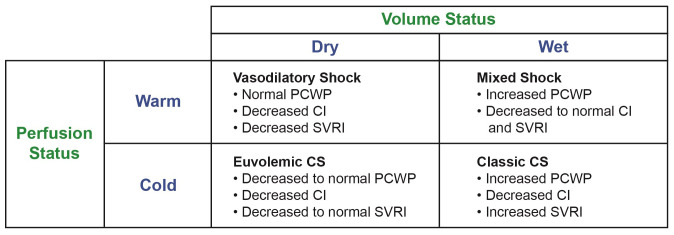
Volume and perfusion profile^26^. CI, cardiac index; CS, cardiogenic shock; PCWP, pulmonary capillary wedge pressure; SVRI, systemic vascular resistance index.

Clinical evaluation of volume overload helps with diagnosis and treatment of AHF exacerbation. One novel physical exam finding that has correlated well with clinical congestion is “bendopenia”, which is defined as dyspnea starting within 30 seconds of bending forward^22^. The prevalence of bendopenia among patients with AHF is reported to be anywhere between 18 to 49%^23^. The clinical exam has been associated with elevated filling pressures, especially in the presence of low cardiac index. Bendopenia is also associated with worsening functional status. The relief of bendopenia through the hospitalization is associated with a decrease in NPs^24^. This physical exam finding may further help with the initial assessment of patients with AHF. One evolving concept related to the importance of reliable assessments of volume and perfusion status is the recognition of the intricate pathophysiology of deranged hemodynamics on end-organ perfusion, which is a major driver of disease progression^25^. A bedside classification proposed by Forrester and Waters for patients admitted with AHF was expanded by Nohria *et al*.^25,26^. These patients were categorized into four profiles based on signs of volume (“wet” vs. dry”) and perfusion (“cold” vs. “warm”). Patients were categorized into “wet-warm” (that is, congestion with adequate perfusion), “wet-cold” (congestion with hypoperfusion), “dry-cold” (no congestion with hypoperfusion), or “dry-warm” (no congestion or hypoperfusion)^26^ (Figure 1).

According to large-scale multinational European registry data, admission and discharge classification based on congestion/perfusion correlated with high mortality rates of 12.1% for the “wet-cold” profile, 9.1% for “dry-cold”, 3.6% for “wet-warm”, and 2% for “dry-warm”. The 1-year all-cause mortality ranged from 12.1% in “dry-warm” patients to 26.4% in “wet-cold” patients^27^. Therefore, evaluation based on congestion/perfusion at admission and discharge could provide invaluable prognostic evaluation in patients presenting with AHF. However, traditional signs and symptoms have shown limited discriminative ability for congestion assessment^28^. Additionally, efforts for routine hemodynamic stratification for AHF in the clinical setting over the past decade have been hampered by the primary findings from the The Evaluation Study of Congestive Heart Failure and Pulmonary Artery Catheterization Effectiveness (ESCAPE) trial, which showed that the addition of the pulmonary artery catheter (PAC)-guided strategy did not affect overall mortality and hospitalization^29^. Of note patients with significant renal dysfunction (creatinine >3.5mg/dl) and cardiogenic shock requiring inotropes were excluded from the trial, which may have reduced the utility of PACs. The findings of the trial has resulted in less and less PAC utilization in the clinical setting^30^. With many noninvasive strategies (for example, impedance cardiography) being tested, the majority were not as reproducible in diagnosing low cardiac output or optimizing hemodynamics and few had been prospectively tested in randomized clinical trials (RCTs), especially in the AHF setting. With advances in temporary mechanical circulatory support for acute cardiogenic shock and the resurgence of hemodynamic parameters in the new cardiogenic shock classification scheme, there has been a resurgence in PAC-guided strategies^31,32^. Recently published real-world retrospective data showed that utilization of PAC-guided therapy resulted in decreased mortality among patients presenting with cardiogenic shock^33^. Among the whole cohort of these patients, having no PAC assessment was associated with higher in-hospital mortality (adjusted odds ratio [OR] 1.57, 95% confidence interval [CI] 1.06–2.33)^33^. Guidelines recommend invasive hemodynamic assessment amongst AHF patients with recurrent exacerbations, or requiring vasopressor support, or have uncertain volume, or perfusion status, or both (Class 1)^34^. Deciphering the volume and perfusion status could help tailor appropriate therapies to AHF patients in a timely manner.

**Figure 2.**
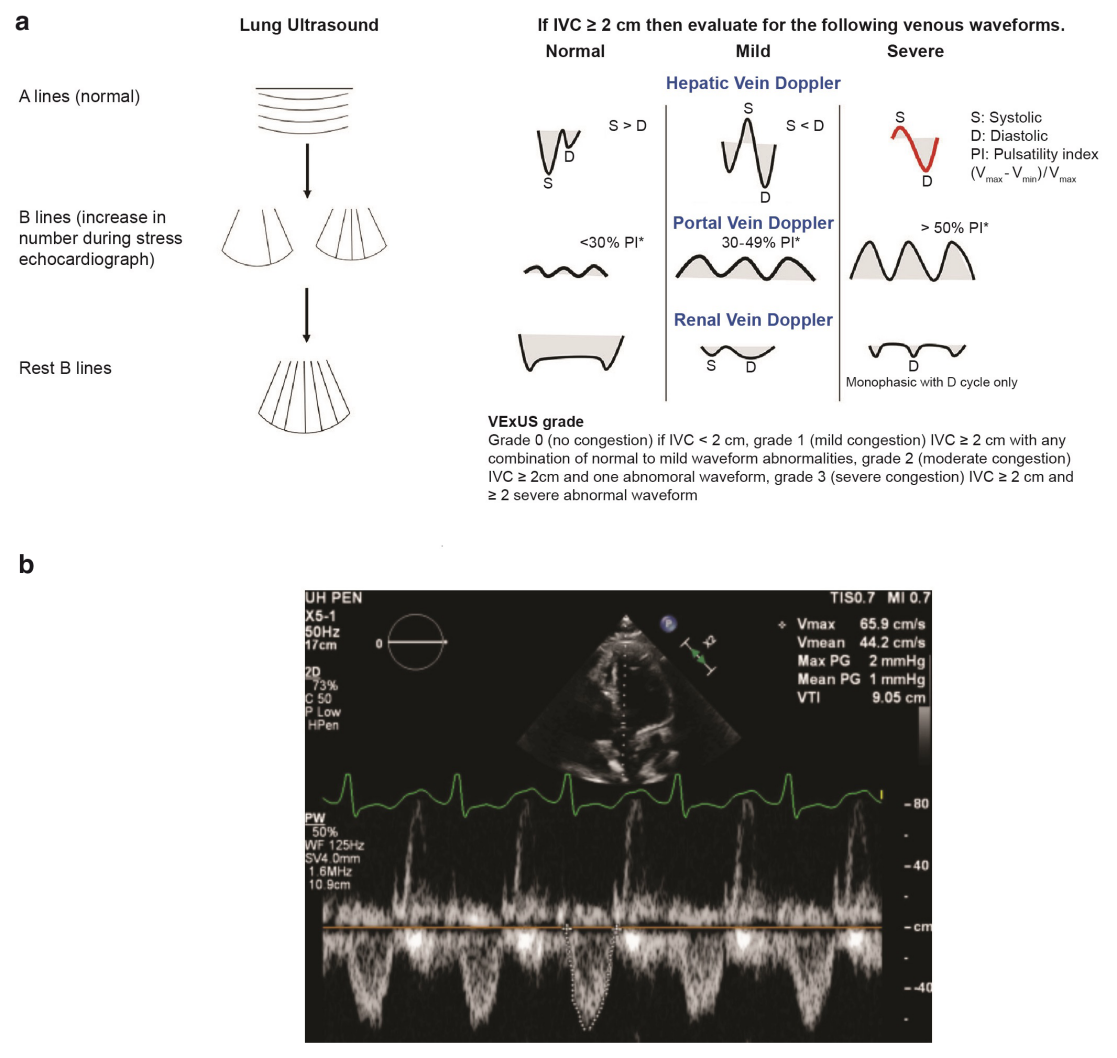
Ultrasound assessment during acute heart failure exacerbation. (**a**) Novel ultrasound assessment of venous congestion and waveforms^39,40^. (**b**) Ultrasound assessment of cardiac function in five-chamber view, showing low left ventricle outflow tract velocity time integral of 9 cm in a patient with acute heart failure. IVC, inferior vena cava; PW, pulsed-wave; VExUS, venous excess ultrasound score; VTI, volume time integral; WF, waveform.

Two promising strategies that have recently evolved to assess congestion involve the use of point-of-care ultrasound technology beyond assessing cardiac structure and function. Central venous congestion, so-called “thoracic comets”, identification of B lines at the thoracic cavity to assess extravascular lung water has gained some popularity^35^. Persistence of B lines prior to discharge is associated with increased incidence of HF readmissions and mortality, therefore routine evaluation upon admission and discharge could guide decongestive strategies and help assess prognostication^36,37^. Extended to broader assessment of venous congestion, inferior vena cava diameter and intrahepatic and intrarenal Doppler flow parameters have also been associated with organ congestion and impaired natriuretic responses to diuretic therapy^38–40^. A physiological study demonstrated that volume expansion in patients with HF irrespective of EF led to significant blunting of venous wavefoms, in setting of elevated central venous pressures, which could lead to increased renal capillary pressure and congestion^40^. Recently, the venous excess ultrasound score (VExUS) was devised on the basis of the ultrasound findings of congestion. It has been reported that dilated inferior vena cava (≥2 cm) along with severe flow abnormalities in intrahepatic and intrarenal vessels outperformed central venous pressures in predicting the risk of acute kidney injury (adjusted hazard ratio [HR] 2.82, 95% CI 1.2–6.6, *P* = 0.02)^41^ (Figure 2a). Along with volume assessment, point-of-care ultrasound can also be used for evaluation of cardiac function in patients with AHF. Left ventricle outflow tract volume time integral (LVOT VTI) is a surrogate marker of stroke volume and is measured in centimeters by placement of pulsed-wave Doppler below the aortic valve in either apical five- or three-chamber view^42^. Studies have shown that low LVOT VTIs are better than EF in predicting mortality and placement of left ventricle assist devices (LVADs), indicating the potential of LVOT VTI in risk-stratifying patients with AHF^43,44^ (Figure 2b). More studies are needed to determine the universal LVOT VTI cutoff to predict the risk of adverse outcomes in patients with AHF. Routine point-of-care ultrasound assessment of patients presenting with AHF may assist in assessing cardiac function, guiding diuretic regimen, and identifying patients at risk of hemodynamic collapse.

Another noninvasive method is the Remote Dielectric Sensing (ReDS) system (Sensible Medical Innovations, Netanya, Israel), which is being explored to quantify pulmonary congestion. It is a miniature radar system which emits electromagnetic signals in a quick reproducible manner that can help provide percentage of fluid content compared with lung volume, and normal values range between 20 to 35%. The device has correlated with computed tomography-measured lung fluid content^45^. A prospective pilot study showed that ReDS technology helped identify about one third of the patients with residual congestion, who were clinically deemed to be decongested and were near discharge^46^. This could help avert HF readmissions. Preliminary primary results from a prospective multicenter study presented in 2019 showed no differences in an intention-to-treat (ITT) analysis but significant reduction in number of HF readmissions in a modified ITT analysis, yet a peer-reviewed manuscript has not been published. An ongoing prospective trial is evaluating ReDS for a SAFE Discharge in Patients with Acutely Decompensated Heart Failure: The ReDS-SAFE HF Study (ClinicalTrials.gov Identifier: NCT04305717).

## How to assess diuretic response

Diuretics are the mainstay therapy for patients presenting with AHF exacerbation. Loop diuretics (LDs) remain the initial choice of management. The Diuretic Strategies in Patients with Acute Decompensated heart failure (DOSE-AHF), a 2×2 factorial trial, evaluated high-dose LDs versus low-dose LDs and bolus LD versus continuous LD infusion. High-dose LD (2.5 times the home dose) in comparison with low-dose LD (home dose) was associated with a statistically significant improvement in dyspnea, weight loss, and negative fluid balance after 72 hours of admission^47^ Worsening renal function (WRF), defined by a rise in creatinine by 0.3 mg/dL at 72 hours, was more frequently reported in the high-dose LD group but compared to stable renal function, was not associated with worse outcomes (HR 1.17, 95% CI 0.77–1.78, *P* = 0.47)^47–49^. Instead improving renal function compared with those who had stable renal function was more likely to have composite outcome of death and/or HF hospitalization (HR 2.52, 95% CI 1.57–4.03, *P* <0.001)^49^. Similarly post-hoc analysis of the Renal Optimization Strategies Evaluation (ROSE) trial, which used high-dose LD for diuresis in AHF patients demonstrated that increase in creatinine or cystatin C was not associated with elevation of urinary renal tubular injury biomarkers such as neutrophil gelatinase-associated lipocalin^50^. These findings indicate that bumps in creatinine in setting of diuresis are associated with benign changes in filtration fraction and are not necessarily indicative of tubular injury. However amongst AHF patients undergoing aggressive diuresis who both had WRF and increase in markers of tubular injury, paradoxically trended towards improved survival (adjusted *P* = 0.045) as compared to patients who had improving renal function and decrease in markers of tubular injury^50^ These studies underscore the importance of not discontinuing diuretics in clinically congested patients, as mild decreases in renal filtration fractions amongst AHF patients receiving decongestive therapies is not associated with renal tubular injury and worsening survival. Instead withdrawal of diuretic therapies in the setting of worsening filtration fraction in clinically congested patients is associated with adverse outcomes. Post-hoc analysis of DOSE-AHF patients showed that patients who received a high dose of LD, when adjusted for diuretic dose, had improved outcomes with decreases in mortality, HF admissions, and hospitalizations (HR 0.64, 95% CI 0.43–0.95, *P* = 0.028). This supports the beneficial role of an aggressive diuretic regimen in AHF patients^51^.

In the DOSE-AHF trial, which also evaluated bolus dosing verus continuous diuretic infusion, continuous infusion did not result in improvement of symptoms and increase in urine output and neither was associated with decrease in HF hospitalizations or mortality. It is important to note that unlike common clinical practice, patients in the diuretic infusion arm did not receive loading dose prior to initiation. Lack of initial bolus dose may have masked the effectiveness of the continuous diuretic infusion. The bolus group more often required escalation of diuretic dose and need for thiazide diuretics as compared with continuous infusion. This led to a non-significant higher cumulative diuretic dosing in the bolus group (592 vs. 480 mg, *P* = 0.06)^47,48^. Diuretic infusions are intended to prevent post-diuretic sodium retention, which is one of the mechanisms of diuretic resistance. A meta-analysis comparing continuous versus bolus dosing did report significant increase in urine output and weight loss without any decrease in mortality with continuous infusion of LDs^52^. The Diuretic Response in Advanced Heart Failure: Bolus Intermittent vs. Continuous Infusion (DRAIN) trial was a prospective RCT, amongst 80 HF patients with reduced EF <30%, which compared infusion versus bolus dosing of LDs. Continuous infusion of LDs was more often associated with relief of congestive symptoms (25% vs. 48%, *P* = 0.04) with significantly increased urine output (10,020 ± 3032 vs. 8612 ± 2984 ml, *P* = 0.04) and less likelihood of treatment failure without significant WRF^53^. Therefore, the continuous infusion of furosemide remains a viable option, especially for AHF patients with refractory hypervolemia.

Further analysis of the DOSE trial also demonstrated differential response to high-dose diuresis based on underlying EF. Amongst patients with HFpEF, high-dose diuresis was associated with significant increase in creatinine (+ 0.16 mg/dL, 95% CI 0.02–0.30 mg/dL, *P* = 0.03), but did not result in net fluid or weight loss or improvement in global congestive symptoms. Comparitvely, patients with HF with reduced ejection (HFrEF) responded well to high-dose diuretics with significant increase in net fluid and weight loss, improvement in congestive symptoms and decrease in HF hospitalizations without increasing creatinine. (− 0.05 mg/dL, 95% CI −0.14–0.03 mg/dL, *P* = 0.23)^54^ The difference in response to diuresis between HFpEF and HFrEF could be secondary to differences in distribution of volume. In a study, it was noted that in response to volume expansion, HFpEF patients as compared to HFrEF patients, were less likely to have intravascular volume expansion and more likely to have interstitial congestion with decreased effective circulating volume^55^. This could make HFpEF patients more sensitive to intravascular volume contraction in setting of diuresis leading to WRF. It is also thought that HFpEF patients are preload-dependent and agressive diuresis can decrease the venous return leading to decrease in left ventricle stroke volume, resulting in decreased renal perfusion. To further investigate this a single center Randomized Evaluation of Heart Failure with Preserved Ejection Fraction Patients with Acute Heart Failure and Dopamine (ROPA-DOP) trial was conducted which showed addition of low-dose dopamine did not significantly decrease incidence of WRF and was associated with a non-significant trend towards increase of diuresis. Additionally compared to bolus dosing, continuous diuretic infusion was significantly associated with WRF (OR 4.32, 95% CI 1.26–14.74, *P* = 0.02)^56^. These findings suggest that amongst HFpEF patients low-dose bolus dosing may be more effective than continuous infusion. More studies are needed to investigate effective diuretic strategies in decompensated HFpEF patients.

**Figure 3.**
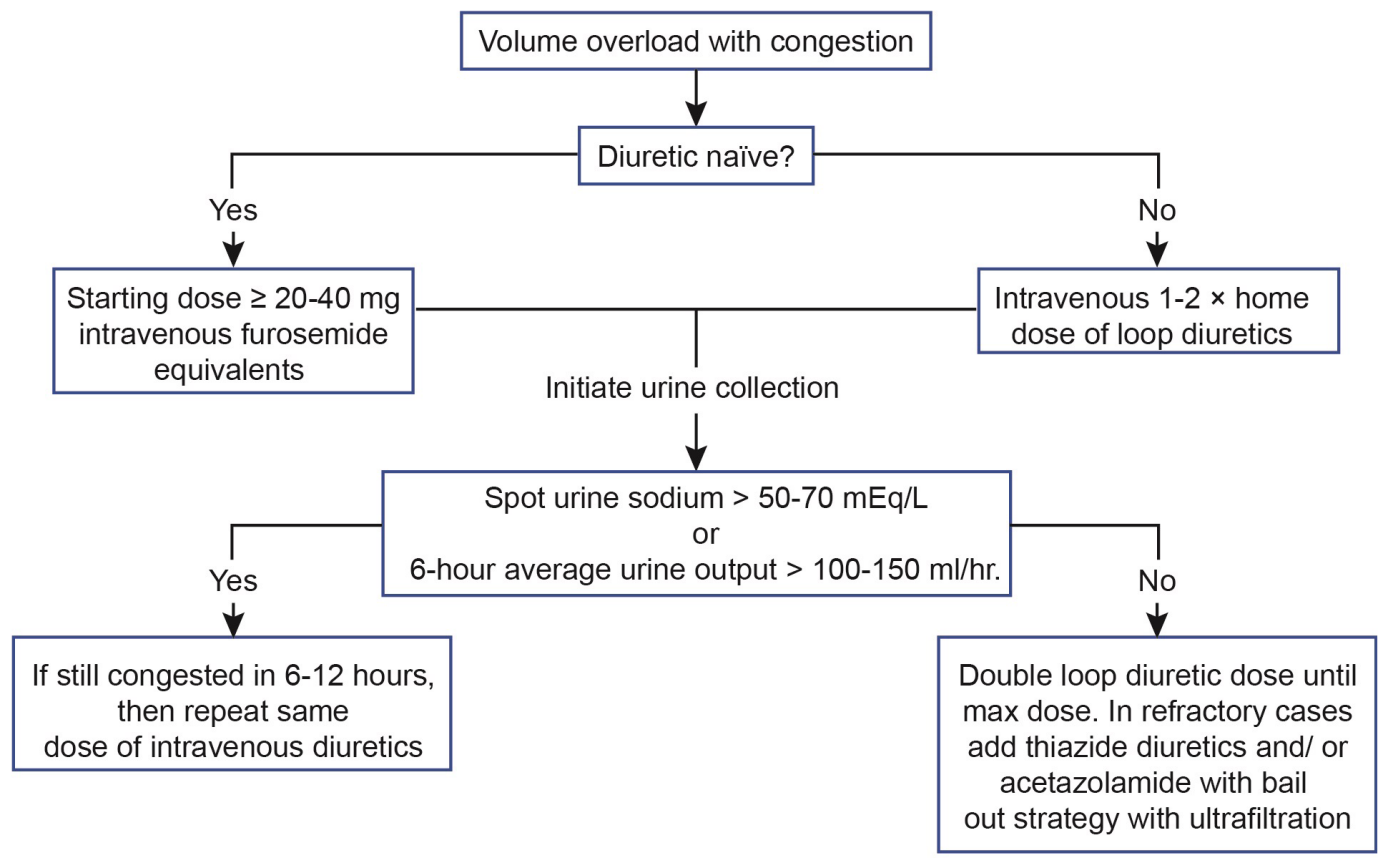
Approach to diuresis among patients presenting with acute heart failure^48^.

The European Society of Cardiology 2021 guidelines recommend the utility of spot urinary sodium or volume of diuresis (or both) to assess diuretic response^57^. Assessing spot urine sodium 2 hours after diuretic administration and checking average hourly urine output after 6 hours of diuretic administration are recommended to assess diuretic response. A spot urine sodium of less than 50 to 70 mEq/L or an hourly urine output of less than 100 to 150 mL/hour is associated with insufficient diuretic response^48^ (Figure 3). A recent publication showed that lower urinary sodium after 6 hours of diuresis was associated with lower urine output on the first day and was an independent predictor of all-cause mortality (HR 3.81, 95% CI 1.92–7.57, *P* <0.001)^58^. Therefore, spot urine sodium check after initiation of diuresis is increasingly recognized as an early marker for diuretic response and an independent prognostic marker for all-cause mortality in patients with AHF^48,58^. A daily dose of 400 to 600 mg furosemide and 10 to 15 mg bumetanide is the maximal daily dose of LD that could be administered^48^. Previously, an open-labeled RCT showed that HF outpatients who received torsemide as compared with furosemide had a decrease in HF hospitalization by 15% with significant improvement in symptomology^59^. The TRANSFORM-HF trial, a large-scale RCT, is under way to compare the effectiveness of torsemide against furosemide in patients with AHF (ClinicalTrials.gov Identifier: NCT03296813).

Limited studies have evaluated the role of subcutaneous diuretics for the outpatient management of worsening HF in an attempt to avert hospitalization. A small phase II study showed that subcutaneous and intravenous (IV) furosemide were equally effective in diuresis and weight change^60^. However, a systemic review of various studies evaluating outpatient subcutaneous and IV furosemide did report a higher admission rate among patients receiving subcutaneous furosemide^61^. Further prospective studies are needed to evaluate the role of subcutaneous diuretics in managing AHF exacerbation in outpatient setting. Recently, the US Food and Drug Administration (FDA) approved extended-release torsemide (once-a-day formulation) for patients with HF and persistent edema^62^. The formulation results in prolonged drug levels in urine, which has been shown to improve natriuresis by preventing post-diuretic sodium retention^63^.

## Diuretic resistance

A recent analysis observed that two thirds of patients with AHF did not require any further treatment beyond initial IV diuretic therapy^64^. However, over 30% of patients with AHF do not achieve clinical decongestion upon discharge, which is associated with higher one-year mortality and HF rehospitalizations^65^. Inability to achieve substantial decongestion coupled with decreased natriuresis in spite of escalating dose of LDs is a phenomenon known as diuretic resistance (DR). This is driven by a multitude of factors, including impaired renal perfusion in the setting of low systemic perfusion, decreased renal filtration, neurohumoral activation, increased renal venous, abdominal pressures, and post-diuretic sodium retention^66^. This leads to increased renin–angiotensin–aldosterone system (RAAS) activation and distal tubular sodium uptake. Risk factors for DR include right ventricle dysfunction, chronic kidney disease, and RAAS activation^67^. In such patients, the efficacy of LDs needs to be augmented by other means and alternative pharmacological targets need to be considered.

In patients with DR, there is an increased renal affinity for sodium. Some studies have evaluated the affect of diuresis with the administration of hypertonic saline (HS). The goal is to acquiesce the sodium avid state of the kidney by delivering sodium to suppress neurohumoral activation and increase intravascular volume/refill rate. Various studies have evaluated the efficacy of administering HS among patients admitted with refractory acute decompensated HF. Co-administration of HS therapy with diuretics among patients admitted with refractory AHF has been associated with statistically significant increases in urine output and weight loss (3.1 ± 0.5 kg at 72 h (*P* <0.001), alongside improvement in sodium and creatinine concentrations. No side effects such as worsening in respiratory or neurological status were reported^68^. A prospective randomized trial also demonstrated that HS addition to diuretis amongst AHF patients was associated with decreases in hospitalization rates (18.5% vs. 34.2%, *P* <0.0001) and mortality (12.9% vs. 23.8%, *P* <0.0001)^69^. Despite positive results from observational studies, physicians have been reluctant to adopt HS as an adjunctive therapy because of concerns for volume and salt overload and need for a critical care setting^67–70^. Further RCTs evaluating the efficacy of HS as an adjunctive to diuretics should be undertaken. A RCT is underway evaluating if oral sodium chloride supplementation increases diuretic efficiency in patients admitted with AHF (ClinicalTrials.gov Identifier: NCT04334668).

Another potential therapeutic approach aims to block sodium re-uptake at the site of maximal reabsorption site in the kidneys, namely the proximal convoluted tubule (PCT), where two thirds of the excreted sodium is reabsorbed. Acetazolamide is a carbonic anhydrase inhibitor that blocks sodium bicarbonate reabsorption at the PCT, which in turn suppresses neurohumoral activation by increasing distal delivery of sodium^71^. In a small RCT, Verbrugge *et al*. randomly assigned patients to acetazolamide with bumetanide versus high-dose bumetanide^71^. Diuretic efficiency, defined as natriuresis per LD dose administered, was higher in the acetazolamide group (84 ± 46 vs. 52 ± 42 mmol/mg, *P* = 0.048). There was also a trend toward decrease in all-cause mortality and hospital readmissions but this did not meet statistical significance^71^. The use of acetazolamide may be effective in augmenting diuresis but further comparative studies are required to establish its efficacy. The data from Acetazolamide in Decompensated Heart Failure With Volume OveRload (ADVOR) RCT is pending (ClinicalTrials.gov Identifier: NCT03505788). Thiazide diuretics constitute the most frequently used strategy to augment efficacy of LDs. Thiazide diuretics counteract the effect of increased sodium uptake in the setting of distal tubular hypertrophy, a phenomenon present among patients with prolonged exposure to LDs. Their use is further considered in patients with low glomerular filtration rate (GFR)^71^. Thiazide diuretics can worsen hyponatremia; therefore, sodium levels should be monitored closely in these patients. Not one thiazide diuretic is proven to be superior than the other. Pharmacokinetically, compared with chlorothiazide, metolazone does have a slower absorption and uptake and a prolonged duration of action^65,71^.

Tolvaptan is a vasopressin 2 receptor antagonist that inhibits the action of anti-diuretic hormone at the collecting tubule. The Efficacy of Vasopressin Antagonism in HF Outcome Study with Tolvaptan (EVEREST) trial did not show decreases in long-term mortality outcomes and HF morbidity. Improvements in dyspnea, body weight, and hyponatremia were noted in patients who received tolvaptan compared with placebo^72^. More recently, the Targeting Acute Congestion with Tolvaptan in Congestive heart failure (TACTICS-HF) trial randomly assigned patients with AHF to tolvaptan versus placebo. The results showed increased weight loss and fluid loss at the expense of WRF but failed to show symptom relief of dyspnea^73,74^. A small randomized Comparison of Oral or Intravenous Thiazides vs. tolvaptan in Diuretic Resistant Decompensated HF (3T) trial compared efficacy of metolazone, chlorothiazide, and tolvaptan against each other among patients refractory to high-dose LDs. All three groups showed increased weight loss and enhanced diuretic efficacy with no statistically significant difference between groups. As predicted, compared with metolazone and chlorothiazide, tolvaptan was associated with a decreased effect on serum sodium^75^. Therefore, the use of tolvaptan is reserved for AHF patients undergoing aggressive diuresis with concurrent severe hyponatremia as outlined by recommendations from ACC Foundation/AHA guidelines^76^. However, the expense of the drug, risk of liver injury rarely requiring transplantation and the lack of supportive efficacy data have limited its broad clinical use for primary diuretic purposes, especially with the availability of other strategies.

## Vasoactive drug therapy

Inotropic and vasodilator therapy is used with the goal to increase cardiac output by augmenting cardiac contractility and reducing afterload among patients presenting with decompensated HF. In patients admitted with AHF without hypotension, vasodilators with nitrates (IV nitroglycerin and sodium nitroprusside) are commonly used. Their use is generally considered to be safe in patients presenting with AHF and is associated with symptom improvement. No mortality benefit is reported and routine use has not been recommended^77^. Inotropic therapy is used in patients with a “cold” profile, especially when there is evidence for systemic hypoperfusion in the setting of low cardiac output state. Data from the Acute Decompensated HF National Registry (ADHERE) indicate significantly increased mortality in patients who are given inotropes, dobutamine, or milrinone compared with vasodilator therapy nitroglycerin or nesiritide^78^. The propensity score-adjusted ORs for nesiritide compared with milrinone and dobutamine were 0.59 (95% CI 0.48–0.73, *P* <0.05) and 0.47 (95% CI 0.39–0.56, *P* <0.05), respectively for in-hospital mortality. For nitroglycerin, adjusted ORs for mortality were 0.69 (95% CI 0.53–0.89, *P* <0.05) and 0.46 (95% CI 0.37–0.57, *P* <0.05) compared with milrinone and dobutamine, respectively. Between nesiritide and nitroglycerin, there was no difference in mortality (95% CI 0.77–1.16, *P* = 0.58) but inotropic use was associated with increased risk^78^. Therefore, inotrope use is generally unfavorable and their role is limited in the setting of palliative care in end-stage HF patients, who have limited options for advanced therapies.

In the Acute Study of Clinical Effectiveness of Nesiritide in Decompensated heart failure (ASCEND-HF) trial, the addition of nesiritide to diuretic therapy in patients with AHF did not result in a decrease in mortality or AHF rehospitalization (9.4% vs. 10.1%, respectively; *P* = 0.31) but did increase episodes of hypotension (26.6% vs. 15.3%, respectively; *P* <0.001)^79^. The ROSE-AHF trial showed that adding either low-dose dopamine or low-dose nesiritide to the standard diuretic regimen did not improve decongestion or renal function^80^. Therefore, the addition of low-dose dopamine and nesiritide to improve renal function in patients with AHF is generally not recommended. However, a post-hoc analysis of the ROSE-AHF trial did reveal differential affect of low-dose dobutamine in HFrEF patients compared to HFpEF. Low-dose dobutamine infusion was associated with more urinary output and significant decrease in mortality in HFrEF patients. In contrast, HFpEF patients with dobutamine were more likely to experience adverse outcomes with increased trend towards mortality. The differential response to inotropy is worth further investigation^81^. In the US, production of nesiritide has been discontinued and nesiritide is no longer commercially available. A recently published RCT showed similar potency of milrinone as compared with dobutamine in patients with cardiogenic shock with no difference in mortality, need for mechanical circulatory support and/or renal replacement therapies (relative risk [RR] 0.90, 95% CI, 0.69–1.19, *P* = 0.47)^82^. The efficacy of vasodilator therapy, though limited, can still be used in the setting of AHF refractory to diuresis, but the use of inotropes to augment cardiac function is associated with increased mortality, limiting their therapeutic utility, especially in a prolonged setting.

Vericiguat is one of the novel agents which is an oral soluble guanylate cyclase activator and enhances the effect of nitrous oxide to increase production of cyclic guanosine monophosphate, which in turn modulates cardiac contraction and vasodilatation^83^. In the Vericiguat Global Study in Subjects with Heart Failure with Reduced Ejection Fraction (<45%), the composite outcome of all-cause mortality and HF hospitalization was significantly reduced among patients who received vericiguat compared with placebo (HR 0.90, 95% CI 0.83–0.98, *P* = 0.02) and the benefit was derived primarily by decreases in HF hospitalization^84^. Further analysis of the trial showed 50% increases in CV mortality and HF hospitalizations among patients who were randomly assigned within 3 months of AHF hospitalization compared with those who received vericiguat without a recent hospitalization^85^. This signifies that patients with recent AHF admission are too sick to benefit from vericiguat. The observation of decreased benefit of vericiguat among the sicker patients with HF was further strengthened by post-hoc analysis aimed at reviewing the efficacy of vericiguat on the basis of NT-proBNP at the time of enrollment. Among patients with NT-proBNP of not more than 8,000 pg/mL, the HRs were 0.85 (95% CI 0.76–0.95) for the primary composite outcome, 0.84 (95% CI 0.75–0.95) for HF hospitalization, and 0.84 (95% CI 0.71–0.99) for CV deaths as compared with HR of 1.16 (95% CI 0.94–1.41) for the primary outcome among patients with NT-proBNP of greater than 8,000 pg/mL^86^. Therefore, NP levels beyond risk stratification could help identify patients who would benefit the most from vericiguat therapy.

Omecamtiv, an inotropic agent under investigation, augments myocardial contractility by enhancing actomyosin interaction without increasing intracellular calcium or oxygen demand. In the Acute treatment with Omecamtiv mecarbil to Increase Contractility in Acute Heart Failure (ATOMIC AHF) phase II trial, higher dose of omecamtiv was associated with improved dyspnea relief and a decrease in left ventricle end-systolic dimension^87^. In the Global Approach to Lowering Adverse Cardiac Outcomes through Improving Contractility in Heart Failure (GALACTIC-HF) phase III trial, omecamtiv administration among patients with chronic HF with reduced EF (≤35%) had a significantly decreased composite endpoint of HF hospitalization and mortality (37 vs. 39.1%, HR 0.92, 95% CI 0.86–0.99, *P* = 0.03)^88^. The effect of baseline left ventricular EF was the strongest predictor of omecamtiv among patients, and a nearly 1.8 times greater effect was noted among the lowest (≤22%) compared with the highest (≥33%) EF quartile^89^. Further studies are needed to elicit the potential role of omecamtiv among AHF patients who are presenting with a concurrent low output state.

Serelaxin, a recombinant form of human relaxin 2 peptide, was evaluated in the RELAX-AHF-2 (Relaxin in AHF 2) trial. The results showed that 48-hour infusion of serelaxin was not associated with a decrease in mortality (8.7% vs. 8.9%, respectively, *P* = 0.77) or worsening HF (5.9% vs. 7.7%, respectively, *P* = 0.19)^90^. Ularitide, a chemical synthesized analogue of naturally occurring vasodilator, was also evaluated in the Trial of Ularitide Efficacy and Safety in ACUTE heart failure (TRUE-AHF). Ularitide infusion compared with placebo was associated with decreases in systolic blood pressure (decrease by 3.9 mm Hg at 48 hours compared with the placebo group, *P* <0.001) and NT-proBNP levels (−3816 vs. −2595 pg/mL, *P* <0.001) but this did not correlate with a decrease in CV mortality (21.7% vs. 21.0%, *P* = 0.75)^91^.

Calcium-sensitizing agents (calcitropic agents) such as levosimendan are also available for use as inotropic agents. These agents increase sensitivity of troponin C to calcium in myocardial cells, enhancing inotropic effects without increasing oxygen consumption of the myocardium along with peripheral vasodilation^92^. In two sequential trials (Randomized Evaluation of Intravenous Levosimendan Efficacy I and II), levosimendan infusion of 24 hours compared with placebo was associated with significant improvement in short-term symptoms, decrease in NPs and days in hospital stay. There was a trend towards non-significant increase in mortality alongside significant increase in episodes of hypotension and cardiac arrhythmias. Adverse CV events may be in part due to the study design, which used a loading dose of levosimendan, a practice that has changed since then. This was alongside the addition of other inotropes among patients who were clinically deteriorating despite being on levosimendan, which may have further contributed to adverse CV events^93^. Another prospective RCT, The Survival of Patients With Acute Heart Failure in Need of Intravenous Inotropic Support (SURVIVE), which compared levosimendan with dobutamine in AHF patients with HFrEF (EF 30%) failed to show a decrease in all-cause mortality (26% vs. 28% respectively, HR 0.91, 95% CI, 0.74–1.13, *P* = 0.40)^94^. Several meta-analyses have shown a decrease in mortality and improvements in hemodynamics, symptom relief, and hospitalization rate among patients receiving levosimendan^95,96^. Further RCTs may help elucidate its utility, especially among patients with AHF complicated by cardiogenic shock.

Another calcitropic agent currently under investigation of AHF treatment is istaroxime, which inhibits the Na^+^/K^+ ^pump and results in activation of the sarco/endoplasmic reticulum Ca^2+^-ATPase to increase intracellular calcium and therefore increasing contractility. In a small RCT, 24-hour istaroxime infusion in patients with AHF was associated with improvement in hemodynamic parameters and decreases in heart rate, NT-proBNP, and self-reported dyspnea^97^. The study also reported improvements in cardiac systolic and diastolic function. No increase in the incidence of adverse CV events was reported^97^. A multinational double-blinded RCT, the Safety and Efficacy of Istaroxime for Pre-Cardiogenic Shock (SEISMiC), is under way to evaluate the efficacy of istaroxime in AHF complicated by cardiogenic shock (ClinicalTrials.gov Identifier: NCT04325035). Other agents under investigation for patients with HF are listed in Table 1.

**Table 1.** Selected clinical trials for patients with acute heart failure.

Trial	Intervention in patients with AHF	Outcome
Diuretics		
EVEREST (2007)^72^ N = 4,133	Tolvaptan vs. placebo	NS difference in long-term mortality outcomes and HF morbidity
DOSE-AHF (2011)^47^ N = 308	High vs. low dose of furosemide infusion Bolus vs. continuous furosemide infusion	NS difference in global assessment of HF symptoms or change in creatinine
TACTICS-HF (2017)^73^ N = 257	Tolvaptan vs. placebo	NS difference in dyspnea relief, despite greater weight loss and diuresis, also worsening renal failure
SECRET of CHF (2017)^74^ N = 250	Tolvaptan vs. placebo	NS improvement in early dyspnea relief, however considerable improvement by day 3, significant weight loss
Ultrafiltration therapy		
UNLOAD (2007)^99^ N = 200	UF vs. loop diuretics	Significant weight and fluid loss (UF arm)
CARESS HF (2012)^100^ N = 188	UF vs. stepped pharmacological diuretics	Significant risk of acute renal failure and adverse events (UF arm) with no difference in weight loss, mortality, or HF hospitalization
AVOID HF (2016)^101^ N = 214	Adjustable UF vs. Adjustable diuretics	Significant decrease in HF rehospitalization and CV events with increase in adverse events (UF arm)
Vasoactive therapy		
SURVIVE (2007)^94^ N = 1,327	Levosimendan vs. dobutamine	NS change in dyspnea relief, all-cause mortality with significant decrease in NPs
PROTECT (2010)^102^ N = 2,033	Rolofylline vs. placebo	NS difference in worsening HF, renal failure, or all- cause mortality
ASCEND-HF (2011)^79^ N = 7,141	Nesiritide vs. placebo	NS different in dyspnea, HF hospitalization, or mortality
REVIVE 1&2 (2013)^92^ N = 600	Levosimendan vs. placebo	Improvement in clinical symptoms, with more frequent hypotension and cardiac arrhythmias and a numerically high risk of death
ROSE-AHF (2013)^80^ N = 360	Low-dose nesiritide vs. low-dose dopamine vs. placebo	NS difference in diuresis or changes in markers of acute renal injury
ATOMIC-AHF (2016)^87^ N = 606	Omecamtiv mecarbil vs. placebo	NS improvement in dyspnea except for patients who received high dose of omecamtiv
TRUE-AHF (2017)^91^ N = 2,157	Ularitide vs. placebo	NS difference in CV death and hierarchical clinical outcomes, significant improvement in NP, and also increase in creatinine
BLAST-AHF (2017)^103^ N = 621	TRV027 vs. placebo	NS difference in all-cause mortality or HF rehospitalization
RELAX-AHF-2 (2019)^90^ N = 6,545	Serelaxin vs. placebo	NS difference in CV death or HF hospitalization
DOREMI (2021)^82^ N = 192	Dobutamine vs. milrinone	NS difference in all-cause mortality
Initiation of goal-directed medical therapy		
ATHENA-HF^104^ (2017) N = 360	Spironolactone (high dose) vs. placebo	NS difference in NPs, mortality, or HF hospitalizations
SOLOLIST-WHF^105^ (2021) N = 1,222	Sotagliflozin vs. placebo	Significant decreases in HF hospitalizations and CV deaths
Iron repletion		
AFFIRM-AHF^106^ (2020) N = 1,108	Intravenous ferric carboxymaltose vs. placebo	Significant decrease in HF hospitalizations, with NS difference in mortality

AHF, acute heart failure; CV, cardiovascular; HF, heart failure; NP, natriuretic peptide; NS, non-significant; UF, ultrafiltration

## Ultrafiltration therapy

A significant proportion of patients with AHF develop cardiorenal syndrome and become refractory to aggressive diuretic management. Ultrafiltration (UF) is the physical removal of isotonic plasma from the patient^98^. In the UF Versus Intravenous Diuretics for Patients Hospitalized for Acute Decompensated heart failure (UNLOAD) trial, 200 patients were randomly assigned to UF or LD. UF was superior to LDs in weight loss (5 ± 3.1 kg vs. 3.1 ± 3.5 kg, respectively; *P*<0.001) and net fluid removal at 48 hours (4.6 ± 2.1 L vs. 3.3 ± 2.6 L, respectively; *P *<0.001), and HF rehospitalization was significantly lower with UF (22 ± 54% vs. 46 ± 76%, respectively; *P* = 0.022)^99^. However, in a follow-up Cardiorenal Rescue Study in Acute Decompensated heart failure (CARRESS-HF) trial, 188 patients were randomly assigned to UF or stepped pharmacological therapy. UF was inferior to stepped pharmacological therapy as there was a significant rise in mean creatinine in the UF arm (+0.23 ± 0.70 mg/dL vs. −0.04 ± 0.53 mg/ dL, respectively; *P* = 0.03) without a significant difference in weight loss (−5.7 ± 3.9 kg vs. −5.5 ± 5.1 kg, respectively; *P* = 0.58). There was no change in 60-day mortality in UF versus the stepped pharmacological group (17% vs. 12%, respectively; *P* = 0.47) and no decrease in composite rate of mortality or HF rehospitalization (38% and 35%, respectively; *P* = 0.96). There was a significant risk of adverse events, including higher incidence of renal failure, IV catheter-related complications, and bleeding complications (72% vs. 57%, *P* = 0.03)^100^. Of note, in the UF arm, there was fixed rate of fluid removal rather than adjustable rate of fluid removal determined by hemodynamics of patients. This may have predisposed patients to adverse clinical outcomes in the UF arm given the increased risk of hypotension^100^.

A later study named Aquapheresis Versus Intravenous Diuretics and Hospitalizations for heart failure (AVOID HF) compared outcomes among AHF patients who received either adjustable UF or adjustable diuretics. The trial was stopped prematurely because of slower-than-expected enrollment. The average rate of UF was 138 mL/hour, which was lower than the fixed rate of 200 mL/hour used in the CARRESS-HF trial. Patients in the adjustable UF arm compared with adjustable diuretics had a non-significant longer time to first HF readmission (62 vs. 34 days, respectively; *P* = 0.10) and a similar 90-day mortality (15% vs. 13%, *P* = 0.87). In regard to secondary outcomes in the UF arm, there was a significant decrease in the number of patients with HF rehospitalization (9.5% vs. 20.4%, respectively; *P* = 0.034) and fewer patients were admitted with CV events (14.3% vs. 25%, respectively; *P* = 0.042). More patients in the UF arm experienced adverse events of special interest, such as central line-associated bloodstream infections, bleeding requiring transfusion, symptomatic hypotension necessitating intervention, and acute coronary syndrome (31% vs. 17%, respectively; *P* = 0.018)^101^. UF is reserved for patients who have AHF refractory to aggressive diuretic management. Another UF modality with a potentially milder impact on hemodynamic shifts among patients with HF is peritoneal dialysis. One study linked peritoneal dialysis use among patients with refractory HF to improved New York Heart Association (NYHA) classification and a significant decrease in the rate of HF hospitalizations^107^. Further studies comparing UF modalities among patients with HF refractory to diuretics are needed.

## Medical therapies after acute heart failure stabilization

In recent years, there have been several advances in disease-modifying pharmacotherapy for patients with HF. With the advent of newer drugs such as ARNI and sodium-glucose co-transporter 2 (SGLT-2) inhibitors, providers now have more tools to combat adverse outcomes in HF. Over time, quadruple therapy with ARNI, SGLT-2 inhibitors, beta blockers, and mineral receptor antagonists has repeatedly been shown to be effective in significantly reducing mortality and HF readmissions^108,109^. The benefit could be seen within days to weeks of starting these medications as event curves diverge quite early in the course of treatment. However, owing to concerns for hypotension, WRF, or hyperkalemia, providers are reluctant to initiate ARNI or mineral receptor antagonist (MRA) in patients admitted with AHF^110^. Simultaneous initiation of these medications may enhance tolerance. ARNI, when compared with enalapril, decreased the risk of hyperkalemia, which thus less likely resulted in MRA discontinuation^111^. Initiation of quadruple therapy could be started at low doses and with slow uptitration. SGLT-2 inhibitors and MRA have minimal to no effect on systolic blood pressure in studies and therefore may be better tolerated^109^. Further studies are needed to evaluate tolerance of initiating these medications together along with the possibility of polypill. Studies have shown that the failure to initiate goal-directed medical therapy (GDMT) during hospitalization often results in less likelihood of outpatient initiation or post-discharge adherence^109,112^.

In the PARADIGM-HF study, among patients with HFrEF, sacubitril–valsartan initiation was compared with enalapril, and there was less primary composite outcome of CV death or HF hospitalization in the ARNI group (21.8% vs. 26.5%, respectively; *P* <0.001)^113^. In the PIONEER (Comparison of Sacubitril–Valsartan versus enalapril in hospital initiation among patients stabilized from an AHF Episode) trial, ARNI initiation was associated with much greater reduction in NT-proBNP levels (−46.7% vs. −25.3%, respectively; *P* <0.001), and a significant effect was seen as early as within the first week. ARNI was well tolerated clinically with no significant increased risk of renal dysfunction, electrolyte disturbances, and incidence of hypotension^114^. Further PIONEER analysis showed that compared to enalapril, ARNI initiation within 8 weeks in stabilized AHF patients, resulted in significant decrease in composite endpoint of death from any cause, HF rehospitalization, LVAD implantation, or cardiac transplant listing (16.3% vs. 9.8%, respectively; *P* = 0.005)^115^. A retrospective analysis also evaluated the initiation of ARNI after stabilization of 22 patients admitted with cardiogenic shock (EF <40%) to a cardiac intensive care unit. After patients had been titrated off inotropes and were not on vasodilators, ARNI therapy was initiated. After initiation, patients had improvement in cardiac output and decreases in pulmonary and filling pressures. No hyperkalemia and mortality were noted in the analysis; 4.5% had acute kidney injury and 18.2% had discontinuation due to hypotension, and half of the latter patients resumed ARNI later. This analysis further reinforced that earlier initiation of ARNI after stabilization of patients with cardiogenic shock and AHF was safe and potentially beneficial^116^.

ARNI use compared with valsartan in HF patients with preserved EF (≥45%) (PARAGON) trial barely missed the composite outcome of decrease in CV mortality and HF hospitalization (894 vs. 1,009 events, respectively, RR 0.87, CI 0.75–1.01, *P* = 0.059)^117^. Further analysis suggested significant clinical benefit among women and patients with mildly reduced to low-normal EF below the median of 57%^118^. A post-hoc analysis of the PARAGON trial showed that patients who had ARNI, as compared with valsartan, initiated within 30 days of previous HF hospitalization had significantly reduced primary composite outcomes (RR 0.73, 95% CI 0.53–0.99) compared with patients who were never hospitalized (RR 1.00, 95% CI 0.80–1.2)^119^. A study in AHF patients with preserved left ventricular EF (PARAGLIDE-HFpEF) is ongoing (ClinicalTrials.gov Identifier: NCT03988634). The FDA recently expanded the indication for ARNI therapy among patients with below-normal EF^120^. The role of ARNI initiation after myocardial infarction was compared with ramipril in the PARADISE-MI trial and results were presented in ACC 2021. Compared to ramapril, sacubitril–valsartan was associated with numerical decreases in primary composite outcome of CV death, first HF hospitalization or outpatient HF (13.2% vs. 11.9% respectively; *P* = 0.17).^121^. The role of ARNI compared with losartan was also evaluated in a LIFE trial among patients with advanced HF with an EF of not more than 20%, which failed to show significant decreases in CV events and HF exacerbations^122^. This indicates that patients with end-stage HF could have only limited benefit from GDMT and that workup for advanced therapies should be considered.

Spironolactone is an MRA that acts as a neurohormonal antagonist. Its use is well established in patients with HFrEF. The Aldosterone Targeted Neurohormonal Combined with Natriuresis Therapy in Heart Failure (ATHENA-HF) RCT evaluated the effect of high-dose (100 mg) spironolactone compared with low-dose (25 mg) spironolactone or placebo in patients with AHF. At 96 hours, there was no significant difference in NT-proBNP or increase in urine output in the high-dose spironolactone arm. There was also no significant decrease in 30-day mortality or time to first HF rehospitalization or emergency visit between patients receiving two doses of spironolactone (HR 1.22, 95% CI 0.68–2.19, *P* = 0.50). At 96 hours, initiation of high-dose spironolactone was well tolerated and there was no risk of hyperkalemia (only one patient in the low-dose spironolactone group had K^+^ 5.5–5.9 mmol/L and no patient in the high-dose spironolactone group had hyperkalemia) or WRF (28% in high-dose spironolactone vs. 32% low-dose spironolactone group*, P* = 0.42). The safety profile cannot be extrapolated to patients with reduced GFR of less than 30 mL/min, as these patients were excluded from the trial^104^. Further analysis of the ATHENA-HF study showed that the levels of spironolactone metabolites were lower than expected in the high-dose spironolactone arm group, which may explain the lack of therapeutic benefit in the high-dose group^123^. Recently, benefits of SGLT-2 inhibitors have provided further consideration to its initiation in an acute setting during an HF exacerbation. Results from the Effect of Sotagliflozin on CV Events in Patients With Type 2 Diabetes Post Worsening Heart Failure (SOLOIST-WHF) trial that enrolled 1,222 hospitalized patients with type 2 diabetes mellitus and recent worsening HF showed a 33% reduction in the primary endpoint of total occurrences of CV deaths, hospitalizations for HF, and urgent visits for HF^105^. The benefit extended to HFpEF patients with AHF (HR 0.48, 95% CI 0.27–0.86, *P* <0.05), which comprised 20% of the whole SOLOIST-WHF trial^105^. Recently, EMPEROR-Preserved, a prospective RCT, showed that empagliflozin use among AHF patients with EF of at least 40% significantly reduced primary composite outcome of CV death or HF hospitalization by 3.3% (HR 0.79, 95% CI 0.69–0.90 *P* <0.001), irrespective of the presence or absence of diabetes^124^. SGLT-2 inhibitors have emerged as an effective pharmaceutical intervention during AHF exacerbation regardless of the underlying EF. Initiation of ARNI and SGLT-2 inhibitor have been associated with initial decline in renal function, however their long-term use was notably associated with improved CV outcomes and decreased progression of chronic kidney disease^125,126^. Therefore mild worsening of renal function during initial course of treatment should not deter the use of disease altering treatment.

The role of beta blockers in HF management is well established. Beta-blocker discontinuation is typically considered in AHF patients presenting with signs of the “wet-cold” hemodynamic profile, given concerns for hypoperfusion. A meta-analysis evaluating discontinuation of beta blockers in AHF showed increased in-hospital mortality (risk ratio 3.72, 95% CI 1.51–9.14), short-term mortality (RR 1.61, 95% CI 1.04–2.49), and combined endpoint of short-term rehospitalization or mortality (RR 1.59, 95% CI 1.03–2.45)^127^. Therefore, if a patient does not have signs of hypoperfusion on presentation, beta-blocker therapy should be continued. Ivabradine is a novel funny sodium channel blocker that results in negative chronotropy, decreasing myocardial stress. Along with GDMT, ivabradine addition in HFrEF patients with sinus rhythm (heart rate >70 bpm) on maximal tolerated beta-blocker therapy has been associated with decreases in composite HF mortality and hospitalization (24% vs. 29%, respectively; *P* <0.001), driven primarily by decrease in HF hospitalization^128^. The small randomized trial ETHIC-AHF showed statistically significant improvement in EF and a decrease in NPs at 4 months and no decrease in mortality or HF hospitalization^129^.

Iron deficiency is associated with adverse clinical outcomes in patients with HF. It results in mitochondrial dysfunction, increased oxidative stress, and reduced myocardial efficiency. Oral iron supplementation has mostly been found to be ineffective given poor gastrointestinal absorption and tolerability. Trials with oral supplementation in HF have been negative. The role of intravenous iron supplementation has been well advised in chronic HF patients with reduced EF (<50%) given favorable results from several RCTs^130^. IV iron supplementation in patients with chronic HF is associated with improved quality of life and decreases in all-cause mortality and HF hospitalization^130^. A recently conducted RCT evaluating IV ferric carboxymaltose in AHF patients with iron deficiency (ferritin <100 μg/L or 100–299 μg/L with transferrin saturation <20%) and reduced EF of less than 50% (AFFIRM-AHF) showed a significant decrease in HF hospitalization during a 52-week follow-up (RR 0.74, 95% CI 0.58–0.94, *P* = 0.013), with no difference in mortality^106^.

## Transition of care

The patient’s clinical trajectory and priorities should determine effective transition of care. Patients who cannot achieve clinical decongestion or are unable to tolerate GDMT with WRF and signs of hypoperfusion should be considered for evaluation of advanced therapies. This entails candidacy for LVAD or heart transplantation. Patients with worsening HF who are not optimal candidates for advanced therapies should be evaluated for palliative care to discuss prognosis and clinically informed decision making. The addition of palliative care to conventional HF management results in quality-of-life improvement and decreases in anxiety and depression. Palliative care should not be confused with hospice care; the former entails discussing what is important to the patient in light of current clinical trajectory^131^. 

Patients who have recovered from AHF and have tolerated GDMT initiation should be prepared for discharge. A multidisciplinary team approach involving input from an experienced physician, nurses, social worker, physical therapist, and nutritionist should be in place for patients being discharged. Modifiable risk factors, which led to initial AHF decompensation, such as dietary intake, medication non-compliance, or missing physician appointments, should be addressed prior to discharge. Barriers to achieve optimal dietary intake or medication non-compliance due to lack of insurance coverage should be identified and addressed. Patient and family education should be instituted to discuss HF symptoms and prognosis, changes in medical regimen, and how to avail optimal care in a timely manner. Follow-up with an HF provider should be arranged within a few days after discharge. A concise summary of the hospitalization events should be documented and available to the outpatient team for review, such as to better respond if a patient calls back for symptom review or is readmitted with AHF^132^.

The need for physical rehabilitation among patients about to be discharged should be evaluated by trained physical therapists. Recently, a single-blind RCT, Rehabilitation Therapy in Older Acute Heart Failure Patients (REHAB-HF), showed promising results. Among frail elderly patients discharged after AHF hospitalization, a tailored transitional approach to physical rehabilitation compared with placebo resulted in improved primary outcome of short physical performance battery scores (composed of standing balance test, a gait-speed [4-m walk] test, and a strength test [as assessed by the time needed to rise from a chair five times]) at 3 months (mean between-group difference 1.5, 95% CI 0.9–2.0, *P* <0.001). Improvements in geriatric depression and functional status were also noted but this did not result in a decrease in rehospitalizations or mortality^133^.

It is standard of care to offer an outpatient discharge appointment to patients being discharged after AHF hospitalization. Over the last year, to limit the spread of coronavirus-19 disease (COVID-19), there has been a surge in virtual visits. Follow-up visits are essential for GDMT uptitration to target doses and to recognize patients at risk of clinical decompensation^134^. In an RCT evaluating outcomes between virtual visits and in-person visits, no differences in composite (log-rank *P* = *0*.18) or individual (*P* = 0.14) components of hospital readmission, emergency room visit (*P* = 0.52), or death (*P* = 0.61) were noted^135^. Further studies are needed to investigate the safety and feasibility of GDMT titration and clinical outcomes in HF patients being evaluated in virtual visits.

**Figure 4.**
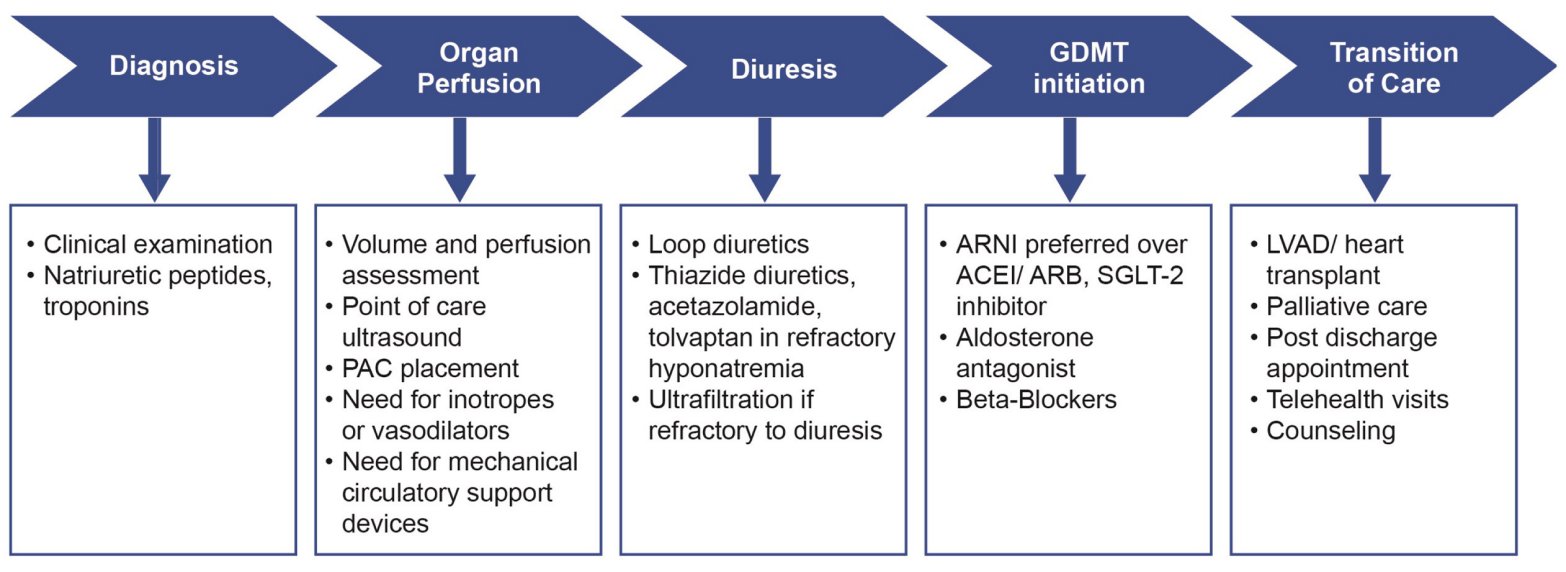
Key goals of acute heart failure management. ACEI, angiotensin-converting enzyme inhibitor; ARB, angiotensin receptor blocker; ARNI, angiotensin receptor-neprilysin inhibitor; GDMT, goal-directed medical therapy; LVAD, left ventricle assist device; PAC, pulmonary artery catheter; SGLT-2, sodium glucose type 2.

Among patients discharged after AHF hospitalization, noninvasive tele-monitoring systems, through daily monitoring of HF symptoms and weight changes, failed to demonstrate a reduction in rehospitalization rates^136^. Wireless direct pulmonary artery pressure monitoring by implantation of CardioMEMS has emerged as a potent technique for monitoring changes in hemodynamic parameters, which may precede clinical congestive symptoms. An RCT called the CardioMEMS Heart Sensor Allows Monitoring of Pressure to Improve Outcomes in NYHA functional Class III Heart Failure Patients (CHAMPION) trial showed that patients receiving medical management based on pulmonary artery pressures had a statistically significantly lower rate of HF hospitalization by 39% after an average follow-up of 15 months^137^. Similar outcomes were noted among HFpEF patients in the CHAMPION trial with a significant decrease in HF hospitalization by 50% at 17 months^138^. Moreover, CardioMEMS implantation was associated with a significant decrease in mortality by 57% among patients receiving GDMT, underscoring the importance of a synergistic ambulatory approach to decongestion and neurohormonal blockade^139^. The benefit of CardioMEMS-guided ambulatory therapy was further evaluated in the recent GUIDE-IT (hemodynamic-guided management of patients with NYHA class II-IV HF) trial. Overall results failed to meet the primary outcome of CV mortality and HF. However, when the results were analyzed for patients enrolled before and during the COVID-19 pandemic, the former seemed to have a significant decrease in HF hospitalization after CardioMEMS enrollment (HR 0.72, 95% CI 0.57–0.92, *P* = 0.007). The benefit did not seem to extend to the advanced NYHA class IV HF patient population^140^. Pour-Ghaz *et al.* also showed that 5-year post CardioMEMS implantation, compared with standard therapy, would be more cost-effective and could lead to better quality-adjusted life-year (QALY) among patients with HF^141^. Currently, the data seem to support remote monitoring of congestion through hemodynamic sensors in NYHA class II or III patients. Figure 4 summarizes several aspects of AHF management both during hospitalization and discharge.

## Conclusions

Although there seemed to be paucity of novel therapies that changed the landscape of AHF treatment, there is much progress in our understanding and the strategic needs of bedside assessment and treatment planning, choices, and adjustments in drug regimens during hospitalization and post-discharge management of AHF. The field has now recognized the lack of robust consideration of the presenting AHF phenotype, the diverse and rather poorly defined therapeutic (both cardiac or comorbid) targets, the over-optimistic expectations of long-term benefits of short-term infusions, and the lack of insights into the determinants of poor outcomes after discharge from hospital. Nevertheless, our decade-long pursuit in clinical trials and registries has enlightened us to recognize three key points:

1. Hemodynamics and assessment of volume and perfusion are highly relevant, and efforts to better stratify endophenotypes to guide therapeutic strategies are much needed.
2. Effective diuretic strategies include both delivering adequate diuretic efficiencies and setting appropriate treatment goals.
3. Short-term interventions only stabilize decompensated states, whereas long-term disease-modifying drug therapies should remain the focus of primary therapeutic goals in AHF.
